# Left ventricle myocardial remodeling following septal myectomy in patients with hypertrophic obstructive cardiomyopathy

**DOI:** 10.1016/j.jocmr.2025.101864

**Published:** 2025-02-17

**Authors:** Guanyu Lu, Liqi Cao, Jiehao Ou, Xinyi Luo, Wei Zhu, Zhicheng Du, Jian Liu, Yuelong Yang, Xinyue Zhang, Peijian Wei, Hongxiang Wu, Huiming Guo, Hui Liu

**Affiliations:** a Department of Radiology, Guangdong Provincial People’s Hospital (Guangdong Academy of Medical Sciences), Southern Medical University, Guangzhou, China; b Department of Interventional Diagnosis and Therapy, Beijing Anzhen Hospital, Capital Medical University, Beijing, China; c Department of Cardiac Surgery, Guangdong Cardiovascular Institute, Guangdong Provincial People’s Hospital (Guangdong Academy of Medical Sciences), Southern Medical University, Guangzhou, China; d The Second School of Clinical Medicine, Southern Medical University, Guangzhou, China; e Department of Echocardiography, Guangdong Provincial People’s Hospital (Guangdong Academy of Medical Sciences), Southern Medical University, Guangzhou, China; f Department of Medical Statistics, School of Public Health, Sun Yat-sen University, Guangzhou, China; g School of Medicine, South China University of Technology, Guangzhou, China; h Department of Pediatrics, The First Clinical College, Guangdong Medical University, Zhanjiang, Guangdong, China; i Department of Structural Heart Disease, National Center for Cardiovascular Disease, China & Fuwai Hospital, Chinese Academy of Medical Sciences & Peking Union Medical College, Beijing, China; j Guangdong Provincial Key Laboratory of Artificial Intelligence in Medical Image Analysis and Application, Guangdong Provincial People's Hospital, Guangdong Academy of Medical Sciences, Guangzhou, China

**Keywords:** Hypertrophic obstructive cardiomyopathy, Magnetic resonance imaging, Fibrosis, Septal myectomy

## Abstract

**Background:**

Left ventricular (LV) reverse myocardial remodeling occurs following septal myectomy in hypertrophic obstructive cardiomyopathy (HOCM), but it remains unclear whether diffuse fibrosis is reversible during this period. Extracellular volume fraction (ECV) and indexed extracellular volume (iECV) are important surrogate markers of diffuse myocardial fibrosis. This study aimed to investigate whether diffuse myocardial fibrosis in HOCM can regress after myectomy.

**Methods:**

A prospective cohort study was conducted among patients with HOCM. All subjects underwent clinical assessment (clinical history, 6-min walk test, biochemical analysis), echocardiography, and cardiovascular magnetic resonance preoperatively and 6 months after septal myectomy.

**Results:**

A total of 43 patients (52±14 years, 23 female) were included in the analysis. At 6 months post-myectomy, there were significant within-person decreases in LV mass index (101.0 [82.5–121.0] to 85.8 [66.7–100.0] g/m^2^; p<0.001), indexed cell volume (68.6 [53.2–82.6] mL/m^2^ to 54.0 [4^2^.6–62.0] mL/m^2^; p<0.001) and iECV (26.5 [22.4–30.1] mL/m^2^ to 21.2 [18.7–26.4] mL/m^2^; p<0.001). Conversely, ECV (28.2±3.3% to 30.2±2.8%; p<0.001) and late gadolinium enhancement mass (4.5 [0.2–8.2] g to 8.7 [2.1–12.8] g; p<0.001) increased. These changes were accompanied by improvement of New York Heart Association functional class, 6-min walk test results, N-terminal pro-B-type natriuretic peptide, and high-sensitivity cardiac troponin T.

**Conclusion:**

Six months after septal myectomy, both cellular hypertrophy and diffuse fibrosis are reversible in HOCM, while focal fibrosis does not regress. These changes are accompanied by improvement of exercise parameters and laboratory biomarkers, revealing the plastic nature of diffuse fibrosis in HOCM and its potential as a therapeutic target.

## Graphical abstract

**Figure:**
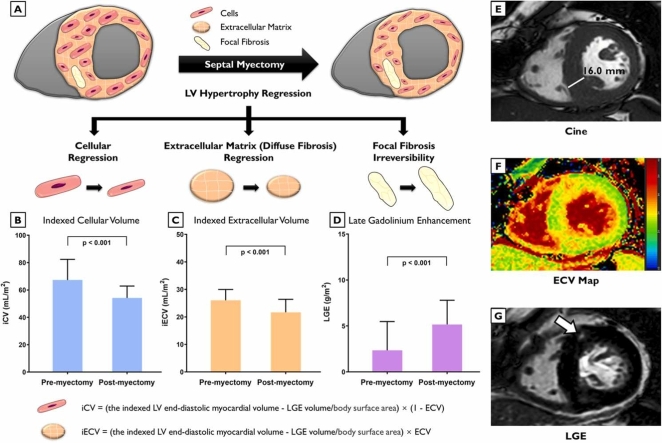
Septal myectomy benefits in cellular hypertrophy and diffuse fibrosis regression. (A) The myocardium consists of cells and the extracellular matrix surrounding them. Diffuse fibrosis is identified by the expansion of the extracellular matrix, while replacement fibrosis occurs following cell death. Cardiovascular magnetic resonance measures LV mass through cine imaging (E), diffuse fibrosis through ECV imaging (F), and focal fibrosis through LGE imaging (arrow) (G). ECV divides the myocardium into cell and matrix compartments, enabling the calculation of cell and extracellular matrix volumes. Septal myectomy benefits in the regression of LV hypertrophy. In this process, both cellular hypertrophy and diffuse fibrosis regress, whereas focal fibrosis is irreversible. Graphics (B), (C) and (D) show comparisons between the pre- and postoperative measures of the iCV, the iECV and LGE, respectively, in hypertrophic obstructive cardiomyopathy. *P*-values indicate differences between measures (significant if <0.05). *LV* left ventricular, *ECV* extracellular volume fraction, *LGE* late gadolinium enhancement, *iCV* indexed cellular volume, *iECV* indexed extracellular volume.

## 1. Background

Hypertrophic obstructive cardiomyopathy (HOCM) is characterized by left ventricular (LV) hypertrophy and left ventricular outflow tract (LVOT) obstruction, which may be caused by genetic or environmental factors [1]. Typical histopathological features include cardiomyocyte hypertrophy, as well as extracellular matrix expansion suggestive of diffuse fibrosis [1]. Surgical septal myectomy has emerged as the preferred treatment for drug-refractory HOCM [2]. This procedure not only immediately eliminates the obstruction, but it has also been shown to lead to progressive LV remodeling after surgery [3], [4], [5]. Previous studies have demonstrated that approximately 1 year following septal myectomy, there was a greater reduction in LV mass compared to the actual mass of the tissue removed during the resection [4], [5], [6]. However, it is currently unknown whether the observed regression is attributed to cellular or interstitial changes, as the determination traditionally necessitated paired myocardial biopsies.

More recently, cardiovascular magnetic resonance (CMR) has emerged as a reliable tool for diagnosing and monitoring LV remodeling due to its excellent myocardial tissue characterization and ability to image in any plane [7], [8]. It can detect focal myocardial fibrosis using late gadolinium enhancement (LGE) [9]. Additionally, T1 mapping technique can non-invasively differentiate between cellular and extracellular compartments and effectively quantify the extracellular matrix expansion [10], [11]. It provides an opportunity to monitor dynamic changes in both cellular and matrix components following septal myectomy. Moreover, extracellular matrix expansion partly reflects an increase in diffuse fibrosis [12]. T1 mapping-derived parameters—extracellular volume fraction (ECV) and indexed extracellular volume (iECV)—have been shown to correlate strongly with diffuse histological fibrosis on myocardial biopsies [10], [11], [12], [13]. ECV quantifies the proportion of extracellular matrix in the myocardium, while iECV reflects the absolute extracellular volume [10], [11], [12], [13]. These markers serve as surrogates for diffuse myocardial fibrosis [10], [11], [12], [13]. Recent studies have further utilized ECV and iECV to track changes in fibrosis over time and responses to interventions, assessing adverse myocardial remodeling and prognosis in conditions such as aortic stenosis [12], [13], [14], [15], [16], [17], [18], [19].

Notably, the outcome of hypertrophic cardiomyopathy (HCM) is not only predicted by the degree of macroscopic LV hypertrophy but also by focal fibrosis and diffuse fibrosis detected using LGE and ECV [20], [21], [22]. Consequently, understanding the microscopic alterations in myocardial structure has the potential to facilitate the identification of more effective treatment targets and a more precise determination of the optimal timing for further intervention. Currently, there are limited data on paired pre- and post-operative CMR markers of myocardial fibrosis for HOCM and postoperative changes in the myocardial structure of HOCM are undetermined [4].

This study aimed to demonstrate pre- to post-septal myectomy changes in myocardial fibrosis in patients with HOCM using CMR. We further explored the potential association between preoperative markers of myocardial fibrosis and the reversal of LV remodeling after surgery.

## 2. Methods

### 2.1 Study population

We conducted a prospective cohort study at a tertiary cardiac center. Patients with definite HOCM and surgical indications for the first septal myectomy were screened between March 2021 and January 2023. The diagnostic criteria for HOCM and the indications for septal myectomy were in accordance with the guideline [5]. Patients were recruited prior to the preoperative evaluation. All subjects underwent preoperative and postoperative clinical assessment (clinical history, 6-min walk test, biochemical analysis), electrocardiography, echocardiography, and CMR. Whole-exome sequencing data were available for 30 patients. Patients with variants in any of the eight HCM-associated core sarcomeric genes (*MYH7*, *MYBPC3*, *TNNT2*, *TNNI3*, *MYL2*, *MYL3*, *TPM1*, and *ACTC1*) deemed pathogenic or likely pathogenic were classified as mutation positive, while those without such variants were classified as mutation negative, according to current guidelines [23]. Postoperative follow-up was conducted at 6 months after septal myectomy. Peripheral venous blood was obtained from each patient within 24 h of CMR for the measurement of high-sensitivity cardiac troponin T (hs-cTNT), N-terminal pro–B-type natriuretic peptide (NT-proBNP), and hematocrit to calculate ECV. Exclusion criteria included age younger than 18 years, pregnancy or breastfeeding, contra-indications to CMR (such as estimated glomerular filtration rate <30 mL/min/1.73 m^2^, allergy to contrast or study medications, and claustrophobia), any evidence of comorbidities (such as congenital heart disease, coronary artery disease, infiltrative cardiomyopathies, valvular disease), abandonment of septal myectomy, mitral valve replacement, absence of routinely postoperative follow-up at 6 months, and suboptimal image quality likely to preclude CMR analysis. Clinical data were collected from the electronic medical records or interviews. The study was conducted in accordance with the Helsinki Declaration. The local Ethics Committee approved the study, protocol, and all subjects gave written informed consent.

### 2.2 CMR protocol

Patients underwent CMR examination within 10 days before and 170 to 190 days after surgery. All CMR studies were performed using a 3.0 Tesla scanner (Ingenia, Philips Medical Systems, Best, the Netherlands) with a 32-channel dedicated phased-array coil. The protocol primarily included steady-state free-precession cine imaging, LGE, and T1 mapping.

ECG-triggered and respiratory navigator-gated balanced steady-state free-precession cine images were acquired in the long-axis plane orientation (2-, 3-, 4-chamber view) and short-axis plane orientation covering both ventricles (no gap). The typical imaging parameters were as follows: field of view (FOV), 250 × 250 mm^2^; voxels, 2 × 2 × 8 mm^3^; repetition time (TR), (3.0–3.2) ms; echo time (TE), (1.5–1.6) ms; flip angle, 45°; slice thickness, 8 mm.

LGE images were obtained 10–12 min after administering a 0.2 mmol/kg intravenous bolus of the contrast agent gadolinium-diethylenetriamine penta-acetic (Consun Pharmaceutical Co., Ltd.) using a phase-sensitive inversion-recovery sequence. The images were acquired in short- and long-axial views, along the same planes as those used for cine images. The acquisition parameters were applied as follows: FOV, 250 × 250 mm^2^; voxels, 2 × 2 × 8 mm^3^; TR, (6.0–6.2) ms; TE, (3.0–3.1) ms; inversion time, adjusted to null the signal intensity of normal myocardium; flip angle, 25°; slice thickness, 8 mm.

Native and post-contrast T1 mapping images were performed using a modified look- locker inversion-recovery sequence in the same basal, midventricular, and apex short-axis planes with “5(3)3″ and “4(1)3(1)2″ scheme, respectively. The post-contrast T1 mapping images were acquired 15–17 min following the administration of the contrast agent. The following acquisition parameters were set: FOV, 250 × 250 mm^2^; TR, 2.4 ms; TE, 1.0 ms; sense factor, 2; minimum inversion time, 105 ms; flip angle, 20°; slice thickness, 8 mm.

### 2.3 CMR analysis

Off-line CMR image analysis was performed using cvi42 image analysis software (Version 5.13.5, Circle Cardiovascular Imaging Inc., Calgary, Canada). The images were analyzed by two experienced radiologists (GY. L and XY. L) who were blinded to clinical information.

Structural and functional analysis of the left ventricle was performed by automatically delineating the LV endocardial and epicardial contours in the short-axis orientation on the end-diastolic and end-systolic phases of the cine images. Manual adjustments were made to the contours whenever necessary to ensure precision. LV volume and LV mass were normalized by body surface area.

LGE being either present or absent was visually evaluated by experienced cardiovascular radiologists. LGE location was assessed using the American Heart Association 16-segment model (excluding the LV apex) [24]. For quantitative analysis of LGE, the high signal intensity region (defined as six standard deviations [SDs] above the mean intensity of the reference region of interest) was automatically contoured, with manual adjustments applied as needed [25]. The percentage of LGE is automatically output by the Tissue Signal Intensity module, calculated as enhanced volume/myocardial volume on the LGE images.

T1 mapping analysis was conducted on the basis of the consensus statement [26]. LV endocardial and epicardial contours were automatically delineated on native T1 maps at three short-axis levels (base, middle, and apex), with manual adjustments made as needed to exclude the blood pool and epicardial fat. Right ventricular insertion points were marked at each short-axis level, resulting in an automatic segmentation into an American Heart Association 16-segment model (excluding the LV apex) [24]. An automatic offset of 10% from the endo- and epicardial edges was applied to minimize partial volume artifacts. T1 values of the blood pool were measured by manually drawing a region of interest within the LV cavity, avoiding the papillary muscles and trabeculae. These delineations were subsequently copied onto corresponding post-contrast T1 maps with stringent adjustments applied to avoid blood pool and artifacts. Native and post-contrast T1 values in each segment were then used to calculate segmental myocardial ECV using the following formula: ECV(%) = (1-hematocrit) × ([1/myo post T1–1/myo nativeT1]/[1/blood post T1–1/blood native T1]). Hematocrit levels were measured within 24 h of CMR scanning in each patient. Segments with LGE (six SDs above the mean intensity of the reference region of interest) on LGE imaging were excluded. The T1 value and ECV for analysis were calculated as the means of all included segments relative to their respective areas. iECV measures the absolute amount of myocardium that is extracellular divided by body surface area. It was calculated as: (the indexed LV end-diastolic myocardial volume–LGE volume/body surface area) × ECV. The indexed cellular volume (iCV) was calculated as: (the indexed LV end-diastolic myocardial volume–LGE volume/body surface area) × (1–ECV). The values are in milliliters per square meter (mL/m^2^).

### 2.4 Cardiac surgery

The detailed septal myectomy procedure (modified and extended Morrow procedure) has been described previously [27]. The operation was performed by a single expert surgeon in the same institution. Intraoperative transesophageal echocardiography was performed to confirm relief of the LVOT obstruction (LVOT gradient <30 mmHg) and absence of moderate or severe mitral regurgitation.

### 2.5 Statistical analysis

Continuous variables are presented as mean±standard deviation or median (interquartile range). Normality was tested using the Shapiro-Wilk test. Categorical variables are expressed as percentages. Preoperative and postoperative within-person changes were compared using a paired Student’s t-test or Wilcoxon signed rank test, as appropriate. Post hoc multiple pairwise comparisons were adjusted with Bonferroni correction. Correlations between the fibrosis variables and changes in cardiac size and function were examined by Pearson correlation coefficient (r). The correlation heatmap was generated with R packages “psych,” “reshape2,” and “ggplot2.” Univariable and multivariable linear regression analyses were conducted using the stepwise method to identify the predictors associated with the reduction in LV mass index (LVMI) and iECV. LVMI, a key marker for assessing myocardial hypertrophy, reflects macroscopic structural changes in the myocardium and is widely recognized as essential for evaluating HOCM in both clinical reports and research [5], [28]. iECV is a surrogate marker for total absolute diffuse myocardial fibrosis, and its variation reflects the absolute change in fibrosis, which is crucial for understanding fibrotic remodeling after myectomy [13], [14]. Only variables with a *P*-value <0.05 by univariable analysis were entered into the multivariable analysis. Residual independence was assessed using the Durbin-Watson test with the “lmtest” package. Outliers or influential points were identified using studentized residuals (>3) or Cook’s Distance (>0.5) with the “car” package. The nonlinear relationships between the change in LVMI or iECV and continuous covariates without linear effects were evaluated using restricted cubic splines with the “splines” and “rms” packages. All statistical analyses were performed using R software (version 4.3.3, R Foundation for Statistical Computing, Vienna, Austria), and GraphPad Prism software (version 9.5.1, California, USA) was used for generating graphs. A 2-sided *P*-value <0.05 was considered statistically significant.

## 3. Results

### 3.1 Study population

**Fig. 1:**
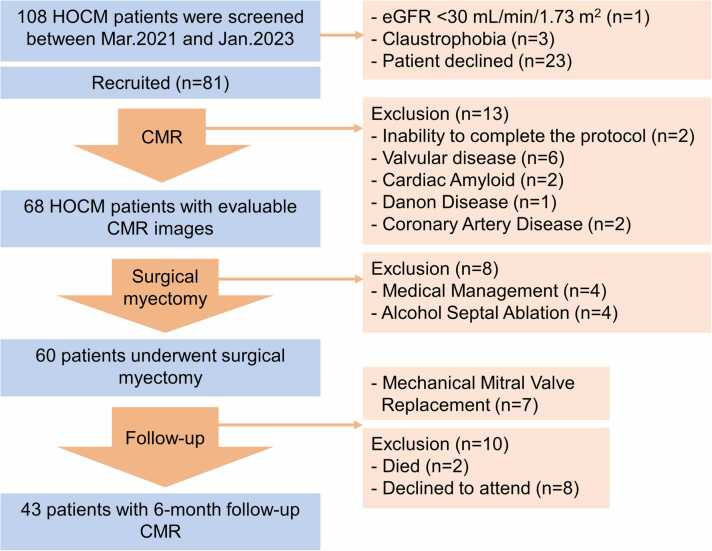
Study flow chart. *HOCM* hypertrophic obstructive cardiomyopathy, *CMR* cardiovascular magnetic resonance, *eGFR* estimated glomerular filtration rate

A total of 108 patients were initially screened for possible inclusion in this study. Four patients with contra-indications to CMR (estimated glomerular filtration rate < 30 mL/min/1.73 m^2^ [n = 1], claustrophobia [n = 3]) were excluded, while 23 patients declined to sign the informed consent forms. Two patients were unable to complete the protocol. Patients with any evidence of comorbidities (valvular disease [n = 6], cardiac amyloid [n = 2], Danon disease [n = 1], coronary artery disease [n = 2]) were excluded. Eight patients did not undergo septal myectomy (medical management [n = 4], alcohol septal ablation [n = 4]), while seven patients who underwent a mechanical mitral valve replacement were excluded after septal myectomy. By 6 months, there were two non-HOCM-related deaths (stroke caused by cerebral arteriovenous malformations [n = 1] and road accident [n = 1]) and 8 patients declined follow-up. Ultimately, 43 patients who underwent 6-month follow-up assessment were included as the study population for analysis (Fig. 1). There was no difference in baseline characteristics between the patients who completed the follow-up and those who did not after surgery (all p>0.05).

The demographic and clinical characteristics of the study population were summarized in Table 1. The mean age at myectomy was 52±14 years old, with 54% (23/43) female. The time from diagnosis to surgery was 12 (1–50) months. The major clinical manifestations of patients were dyspnea (65%, 28/43) and chest tightness (56%, 24/43). At baseline, among 43 patients, 27/43 (63%) received beta-blockers, 10/43 (23%) received calcium channel blockers, 2/43 (5%) were treated with angiotensin-converting-enzyme inhibitors or angiotensin receptor blockers (ARB), 2/43 (5%) were prescribed statins, and 3/43 (7%) underwent amiodarone therapy. At the 6-month follow-up, 20/43 (47%) patients were on beta-blockers, 7/43 (16%) on calcium channel blockers, 4/43 (9%) on amiodarone, 2/43 (5%) on eGFR inhibitors or ARB, and 2/43 (5%) on statins. Of the 30 patients with genetic data, 11/30 (37%) were identified as mutation positive. The actual amount of resection was 7.4±2.7 g (4.4±1.5 g/m^2^). Histological myocyte hypertrophy and disarray were found in all myocardial specimens surgically resected from the 43 patients (Fig. S1). There was no permanent pacemaker implantation and no postoperative ventricular septal defect.

**Table 1:** Demographic and clinical characteristics of study population (N = 43).

Parameter	HOCM Patients (N = 43)
Age, y	52 ± 14
Female, n (%)	23(54)
BSA, m^2^	1.67 ± 0.17
Body mass index, kg/m^2^	24.0 ± 3.2
Heart rate, bpm	66 ± 10
History of tobacco use, n (%)	3(7)
Family history of HCM, n (%)	4(9)
Time from diagnosis to surgery, months	12(1–50)
Comorbidities	
Hypertension, n (%)	12(28)
SBP, mm Hg	128 ± 19
DBP, mm Hg	74 ± 11
Diabetes, n (%)	3(7)
Hyperlipidemia, n (%)	5(12)
Atrial fibrillation, n (%)	2(5)
Symptom	
Dyspnea, n (%)	28(65)
Syncope, n (%)	3(7)
Palpitation, n (%)	13(30)
Chest tightness, n (%)	24(56)
Chest pain, n (%)	17(40)
NYHA functional class III–IV, n (%)	31(72)
Blood	
NT-proBNP, pg/mL	1543(893–2396)
hs-cTNT, pg/mL	22.4(12.4–30.4)
Hematocrit, %	39.3(36.4–43.5)
Echocardiography	
Moderate or severe mitral regurgitation, n (%)	33(77)
SAM of the mitral valve, n (%)	36(84)
Peak LVOT PG, mmHg	87.0(80.0–99.0)
LVOT Vmax, m/s	4.7(4.5–5.0)
Midventricular obstruction, n (%)	6(14)
Medication at baseline	
ACE inhibitor/ARB, n (%)	2(5)
Beta-blocker, n (%)	27(63)
Calcium channel blockers, n (%)	10(23)
Amiodarone, n (%)	3(7)
Statin, n (%)	2(5)
Medication at 6-month follow-up	
ACE inhibitor/ARB, n (%)	2(5)
Beta-blocker, n (%)	20(47)
Calcium channel blockers, n (%)	7(16)
Amiodarone, n (%)	4(9)
Statin, n (%)	2(5)

Values are mean ± standard deviation, n (%), or median (interquartile range).

*ACE* angiotensin-converting-enzyme, *ARB* angiotensin receptor blocker, *BSA* body surface area, *DBP* diastolic blood pressure, *hs-cTnT* high-sensitivity cardiac troponin T, *HCM* hypertrophic cardiomyopathy, *HOCM* hypertrophic obstructive cardiomyopathy, *LVOT* left ventricular outflow tract, *NT-proBNP* N-terminal pro–B-type natriuretic peptide, *NYHA* New York Heart Association, *PG* pressure gradient, *SAM* systolic anterior motion, *SBP* systolic blood pressure, *Vmax* peak velocity, *bpm* beats per minute

### 3.2 Clinical benefits

As shown in Table 2, the proportion of patients with New York Heart Association (NYHA) functional class I–II increased from 28% (12/43) preoperatively to 95% (41/43) at 6-month post-myectomy. The mean 6-minute walk test distance also increased within-person from 473±65 m to 581±74 m (p<0.001). Furthermore, the LVOT pressure gradient showed a significant decrease from 87.0 (80.0–98.5) mmHg to 8.0 (5.0–16.0) mmHg. At baseline, 77% (33/43) of patients had moderate or severe mitral regurgitation, while only 5% (2/43) had this condition during the 6-month follow-up assessment, accompanied by a complete elimination of the mitral valve systolic anterior motion. In terms of clinical chemical laboratory parameters, levels of NT-proBNP and hs-cTnT were reduced within-person (NT-proBNP: 1543 [858–2514] pg/mL to 514 [316–1049] pg/mL; p<0.001, hs-cTnT: 22.4 [12.4–30.4] pg/mL to 15.1 [10.0–22.8] pg/mL; p<0.001).

**Table 2:** Changes after septal myectomy (N = 43).

	Preoperative	Postoperative	Change^a^	p-value
NYHA functional class				
I, n (%)	2(5)	26(60)	+24(56)	-
II, n (%)	10(23)	15(35)	+5(12)	-
III, n (%)	27(63)	2(5)	−25(58)	-
IV, n (%)	4(9)	0	−4(9)	-
6-min walk test, m	473±65	581±74	+108±55	<0.001*
NT-proBNP, pg/mL	1543(858–2514)	514(316–1049)	−816(−1580 to −522)	<0.001*
hs-cTNT, pg/mL	22.4(12.4–30.4)	15.1(10.0–22.8)	−1.9(−8.8 to 0.1)	<0.001*
Complete left bundle branch block, n (%)	2(5)	22(51)	+20(47)	-
Echocardiography				
LVOT PG, mmHg	87.0(80.0–98.5)	8.0(5.0–16.0)	−73.6±23.5	<0.001*
LVOT Vmax, m/s	4.7(4.5–5.0)	1.6(1.1–2.0)	−2.9±1.0	<0.001*
Moderate or severe mitral regurgitation, n (%)	33(77)	2(5)	−31(72)	-
SAM, n (%)	36(84)	0	−36(84)	-
CMR parameters				
LV parameters				
LVEDVI, mL/m^2^	86.6(76.0–94.3)	76.6(68.3–83.8)	−8.2±13.0	<0.001*
LVESVI, mL/m^2^	29.7(25.7–33.5)	28.5(25.0–35.5)	−0.8(−2.6 to 2.2)	0.433
SVI, mL/m^2^	55.6(48.1–62.8)	47.9(41.5–52.8)	−7.7(−14.2 to −4.0)	<0.001*
LVEF, %	65.9(62.2–67.9)	61.8(59.2–65.5)	−2.6±3.1	<0.001*
Cardiac output, L/min	6.5(5.2–7.0)	5.8(4.7–6.3)	−0.7(−1.4 to −0.1)	0.001*
LV mass, g	179.0(138.0–213.0)	137.0(110.0–171.0)	−33.2±14.7	<0.001*
LVMI, g/m^2^	101.0(82.5–121.0)	85.8(66.7–100.0)	−19.8±8.4	<0.001*
LV Maximal wall thickness, mm	23.0(20.0–27.0)	20.0(16.5–23.0)	−3.0(−4.0 to −2.0)	<0.001*
Tissue characterization				
LGE 6 SD method, g	4.5(0.2–8.2)	8.7(2.1–12.8)	+2.9(0.9–5.3)	<0.001*
LGE 6 SD method, g/m^2^	2.7(0.1–5.5)	5.5(1.2–7.7)	+1.8(0.5–3.4)	<0.001*
LGE 6 SD method, %	2.8(0.1–5.9)	6.3(1.6–9.8)	2.9(0.9–4.1)	<0.001*
Native T1, ms	1313±34	1318±34	+5.8±33.3	0.260
ECV, %	28.2±3.3	30.2±2.8	+1.2(0.1–3.2)	<0.001*
iCV, mL/m^2^	68.6(53.2–82.6)	54.0(42.6–62.0)	−16.6±6.6	<0.001*
iECV, mL/m^2^	26.5(22.4–30.1)	21.2(18.7–26.4)	−4.4±2.6	<0.001*

Values are mean±standard deviation, n (%), or median (interquartile range).

*CMR* cardiovascular magnetic resonance, *ECV* extracellular volume fraction, *iCV* indexed cellular volume, *iECV* indexed extracellular volume, *LGE* late gadolinium enhancement, *LV* left ventricular, *LVEDVI* left ventricular end-diastolic volume index, *LVEF* left ventricular ejection fraction, *LVESVI* left ventricular end-systolic volume index, *LVMI* left ventricular mass index, *SD* standard deviation, *SVI* stroke volume index, *ACE* angiotensin-converting-enzyme, *ARB* angiotensin receptor blocker, *BSA* body surface area, *DBP* diastolic blood pressure, *hs-cTnT* high-sensitivity cardiac troponin T, *HCM* hypertrophic cardiomyopathy, *HOCM* hypertrophic obstructive cardiomyopathy, *LVOT* left ventricular outflow tract, *NT-proBNP* N-terminal pro–B-type natriuretic peptide, *NYHA* New York Heart Association, *PG* pressure gradient, *SAM* systolic anterior motion, *SBP* systolic blood pressure, *Vmax* peak velocity

* Indicates a statistical significance after the Bonferroni correction that required a p-value <0.0025 (0.05/20).

a Values for changes presented as mean±standard deviation or median (interquartile range) represent the mean or median of the within-person differences.

### 3.3 Left ventricular remodeling and myocardial fibrosis at 6 months after myectomy

Table 2 depicted the within-person changes in CMR parameters. After 6 months following myectomy, there was a reduction in LV ejection fraction (65.9 [62.2–67.9]% to 61.8 [59.2–65.5]%; p<0.001), which remained in the normal range. Besides, the LV end-diastolic volume index decreased from 86.6 (76.0–94.3) mL/m^2^ to 76.7 (68.3–83.8) mL/m^2^ (p<0.001), while there was no difference between the pre- and postoperative LV end-systolic volume index (29.7 [25.7–33.5] mL/m^2^ to 28.5 [25.0–35.5] mL/m^2^; p = 0.433).

**Fig. 2:**
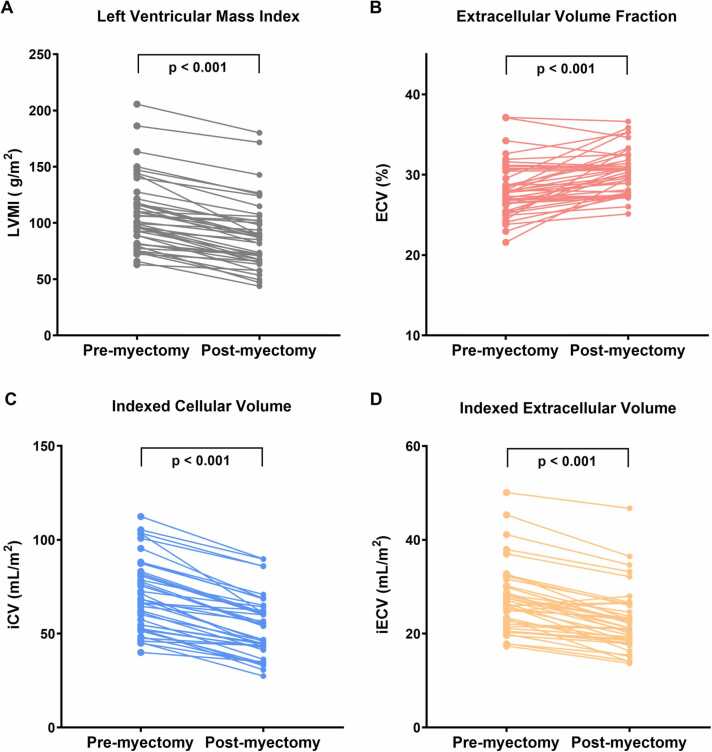
Cell and matrix remodeling 6 months after septal myectomy. Preoperative and postoperative within-person changes in (A) LVMI, (B) extracellular volume fraction, (C) iCV, and (D) indexed extracellular volume. *iCV* indexed cell volume, *LVMI* left ventricular mass index

**Fig. 3:**
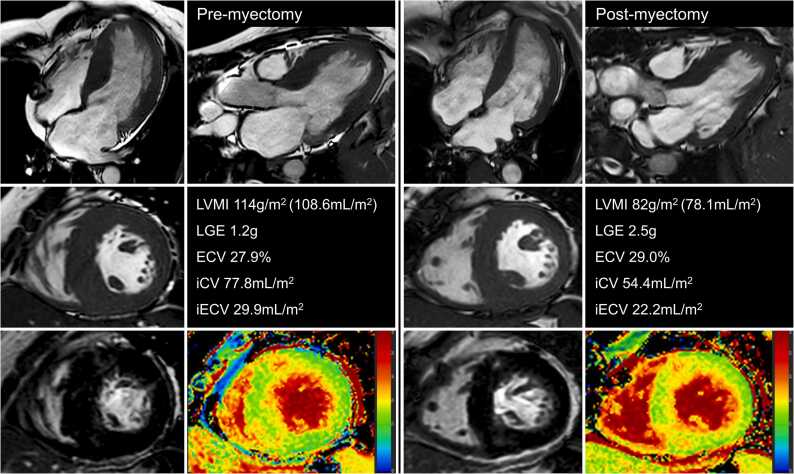
Reverse myocardial remodeling after septal myectomy. A 50-year-old man with hypertrophic obstructive cardiomyopathy (LVOT pressure gradient = 91 mmHg; peak velocity = 4.8 m/s; with systolic anterior motion of the mitral valve). Cardiac magnetic resonance before septal myectomy showed LV hypertrophy (114 g/m^2^). LGE demonstrated focal fibrosis (1.2 g). ECV was 27.9%. At 6 months after septal myectomy, there was a 28% reduction in LV mass (to 82 g/m^2^). The LV mass regression resulted from reductions of cell volume (iCV: 77.8 mL/m^2^ to 54.4 mL/m^2^) and extracellular matrix volume (iECV: 77.8 mL/m^2^ to 54.4 mL/m^2^), so the ECV rose to 29.0%. The focal fibrosis mass increased to 2.5 g. *ECV* extracellular volume fraction, *iCV* indexed cellular volume, *iECV* indexed extracellular volume, *LGE* late gadolinium enhancement, *LV* left ventricular, *LVMI* left ventricular mass index, *LVOT* left ventricular outflow tract

Notably, the results showed LV hypertrophy regression, which was reflected in lower postoperative LV mass (179.0 [138.0–213.0] g to 137.0 [110.0–171.0] g; p<0.001), LVMI (101.0 [82.5–121.0] g/m^2^ to 85.8 [66.7–100.0] g/m^2^; p<0.001) and LV maximal wall thickness (23.0 [20.0–27.0] mm to 20.0 [16.5–23.0] mm; p<0.001). The distribution of LGE in the LV was heterogeneous both pre- and post-myectomy, with LGE predominantly observed at the interventricular septum and the anterior wall at the basal and mid-ventricular levels (Fig. S2). Compared to the preoperative assessment, focal fibrosis (LGE extent) was increased in the 6-month follow-up assessment, both in terms of absolute mass (4.5 [0.2–8.2] g to 8.7 [2.1–12.8] g; p<0.001) and percent of LV mass (2.7 [0.1–5.5]% to 5.5 [1.2–7.7]%; p<0.001). Furthermore, ECV measurements increased from 28.2±3.3% to 30.2±2.8% (p<0.001) with a concomitant decrease in postoperative iCV (68.6 [53.2–82.6] mL/m^2^ to 54.0 [42.6–62.0] mL/m^2^; p<0.001) and iECV (26.5 [22.4–30.1] mL/m^2^ to 21.2 [18.7–26.4] mL/m^2^; p<0.001) (Fig. 2, Fig. 3). There was no difference between pre- and postoperative native T1 values (1313±34 ms vs 1318±34 ms; p = 0.260). The correlations between the fibrosis variables and changes in cardiac size and function are demonstrated in Fig. S3.

**Fig. 4:**
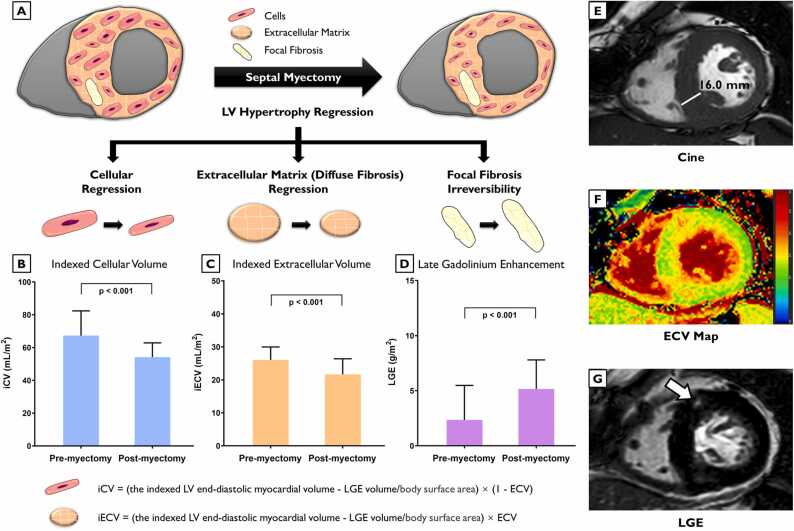
(Graphical Abstract): Septal myectomy benefits in cellular hypertrophy and diffuse fibrosis regression. (A) The myocardium consists of cells and the extracellular matrix surrounding them. Diffuse fibrosis is identified by the expansion of the extracellular matrix, while replacement fibrosis occurs following cell death. Cardiovascular magnetic resonance measures LV mass through cine imaging (E), diffuse fibrosis through ECV imaging (F), and focal fibrosis through LGE imaging (arrow) (G). ECV divides the myocardium into cell and matrix compartments, enabling the calculation of cell and extracellular matrix volumes. Septal myectomy benefits in the regression of LV hypertrophy. In this process, both cellular hypertrophy and diffuse fibrosis regress, whereas focal fibrosis is irreversible. Graphics (B), (C) and (D) show comparisons between the pre- and postoperative measures of the iCV, the iECV and LGE, respectively, in hypertrophic obstructive cardiomyopathy. *p*-values indicate differences between measures. *iCV* indexed cellular volume, *iECV* indexed extracellular volume, *LV* left ventricular, *ECV* extracellular volume fraction, *LGE* late gadolinium enhancement

### 3.4 Factors associated with left ventricle reverse remodeling

In univariable analysis, LVMI regression was associated with baseline LVMI, iCV, and ECV (all p<0.05) (Table 3 and S1). Similarly, baseline iECV and ECV were associated with the absolute value of the change in iECV from preoperative to 6 months postoperatively (both p<0.05) (Table 3 and S2). The results of multivariable analysis showed that baseline iCV (β=0.60, p=0.029) was an independent predictor of the absolute reduction in LVMI (Table 3 and S3). Furthermore, higher baseline iECV (β = 0.14, p = 0.011) and ECV (β = 0.22, p = 0.043) were independent predictors of a greater absolute reduction in iECV from preoperative to 6 months post-myectomy (Table 3). After excluding influential data point, higher baseline ECV (β = 0.27; p = 0.011) and iECV (β = 0.21; p<0.001) remained associated with the reduction in iECV. In the spline analysis, no nonlinear effect was observed in the association between the change in LVMI and the covariates: age, actual amount of resection, native T1, or iECV, nor between the change in iECV and the covariates: age, actual amount of resection, LVMI, LGE, native T1, or iCV (all p>0.05).

**Table 3:** Predictors of change in indexed left ventricular mass and indexed extracellular volume after septal myectomy.

	Univariable Predictors			Multivariable Predictors		
	Beta	95%CI	*P*-value	Beta	95%CI	p-value
Predictors of change in LVMI after Septal Myectomy^a^						
Age, yrs	0.03	−0.15 to 0.22	0.733			
Female	−0.76	−5.98 to 4.45	0.770			
Actual amount of resection, g	0.14	−0.82 to 1.10	0.775			
LVMI, g/m^2^	0.08	0.003 to 0.161	0.041	−0.26	−0.56 to 0.04	0.084
LGE 6 SD method, %	−0.43	−0.89 to 0.03	0.067			
Native T1, ms	−0.07	−0.14 to 0.01	0.079			
ECV, %	−1.13	−1.83 to −0.43	0.002	−0.11	−1.16 to 0.94	0.833
iCV, mL/m^2^	0.20	0.08 to 0.33	0.002	0.60	0.07 to 1.13	0.029
iECV, mL/m^2^	0.25	−0.11 to 0.61	0.161			
Predictors of change in iECV after Septal Myectomy*						
Age, yrs	0.01	−0.05 to 0.07	0.776			
Female	−0.53	−2.15 to 1.09	0.511			
Actual amount of resection, g	0.09	−0.21 to 0.39	0.551			
LVMI, g/m^2^	0.02	−0.003 to 0.047	0.087			
LGE 6 SD method, %	0.02	−0.13 to 0.17	0.805			
Native T1, ms	0.02	−0.01 to 0.04	0.213			
ECV, %	0.27	0.04 to 0.50	0.024	0.22	0.01 to 0.44	0.043
iCV, mL/m^2^	0.03	−0.01 to 0.07	0.180			
iECV, mL/m^2^	0.15	0.05 to 0.26	0.006	0.14	0.03 to 0.24	0.011

Change in LVMI or iECV = preoperative LVMI or iECV − postoperative LVMI or iECV. All predictors reflect baseline (preoperative) data or measurements.

*CI* confidence interval, *CMR* cardiovascular magnetic resonance, *ECV* extracellular volume fraction, *iCV* indexed cellular volume, *iECV* indexed extracellular volume, *LGE* late gadolinium enhancement, *LV* left ventricular, *LVMI* left ventricular mass index, *SD* standard deviation.

* Predictors including ECV and iECV: Durbin-Watson statistic = 2.56, p = 0.972.

a Predictors including LVMI, ECV, and iCV: Durbin-Watson statistic = 2.26, p = 0.817.

## 4. Discussion

In this prospective cohort study, we gained valuable insights into the changes that occurred in cellular and matrix components during reverse myocardial remodeling in patients with HOCM 6 months after surgical septal myectomy. The main findings are as follows: (i) 6 months after myectomy, the reversal of LV hypertrophy is due to the regression of both myocardial cellular hypertrophy and extracellular matrix expansion (diffuse fibrosis). These changes are accompanied by improvement in exercise parameters and laboratory biomarkers; (ii) diffuse fibrosis (iECV) regresses significantly after myectomy, while focal fibrosis (LGE) is not reduced; and (iii) preoperative iCV is associated with LV mass regression, while preoperative iECV and ECV were related to the absolute reduction in iECV.

Our study revealed that 6 months after septal myectomy, there was a decrease in LV mass that exceeded the actual resection of the myocardium. Previous research has shown that LV mass in HCM patients increased progressively over time [20], [29]. Our finding suggests that myectomy not only relieves the progressive increase in LV mass observed in HOCM patients, but also prompts reverse remodeling of the LV myocardium, which is in accordance with the prior studies [4], [6]. The reversibility (in part) of myocardial mass after elimination of the LVOT obstruction implies that hypertrophy in HOCM is, at least in part, a secondary consequence of the dynamic obstruction of the LVOT [6], [28]. ECV represents the relative extracellular volume, while iECV reflects the absolute extracellular volume [15]. Since ECV is also influenced by cellular volume changes, iCV and iECV offer a more comprehensive understanding of remodeling in both cellular and extracellular compartments (Fig. S4) [12]. We observed a decrease in iCV following myectomy. Nagueh et al. demonstrated a significant reduction in myocyte size after the LVOT gradient reduction [30]. In our study, all myocardial specimens showed histological myocyte hypertrophy at baseline. Thus, the decrease in iCV post-myectomy may result from regression of cellular hypertrophy, along with potential reductions in cardiomyocyte number and cellular atrophy. We further demonstrated that not only iCV but also iECV decreased following myectomy, indicating that the regression of cardiomyocytes and the extracellular matrix jointly contribute to the reversal of LV hypertrophy during this period. In contrast, we observed an apparently paradoxical increase in ECV from pre- to postoperative periods. This finding suggests that the postoperative decrease in cellular and extracellular volumes is not balanced and that the cellular components regress proportionately more than the extracellular matrix, consequently leading to an increased ratio of extracellular volume to the total myocardial volume 6 months after myectomy. This finding helps explain the significant association observed between iCV prior to myectomy and LV mass regression. Moreover, these changes were accompanied by the improvement of exercise parameters and laboratory biomarker parameters.

In the present study, we found a decrease in iECV 6 months after myectomy. As iECV correlates well with diffuse histological fibrosis on myocardial biopsies and has been used as a surrogate marker for total absolute diffuse myocardial fibrosis [13], [14], the decrease in iECV reflects a reduction in absolute volume of diffuse myocardial fibrosis during LV remodeling. In other words, this finding suggests that diffuse myocardial fibrosis is potentially reversible for HOCM patients. To the best of our knowledge, this is the first study to elucidate this change in HOCM patients following surgical septal myectomy, although it has been previously validated in myocardial response to therapy for other cardiac diseases [14], [16]. It highlights the dynamic nature and plastic character of the reactive diffuse fibrosis (extracellular matrix), which is influenced by changes in collagen turnover caused by the reaction of fibroblasts to postoperative mechanical and local humoral factors [31]. Indeed, the potential for the degradation of myocardial fibrosis and the reversal of cross-linking continues to attract intense research interest and activity [16]. Our findings suggest that septal myectomy can not only reverse LV hypertrophy at the macro level, but also lead to modification of the microenvironment in the myocardium. In addition, our analysis showed that ECV and iECV were associated with the changes in iECV, which is expected, as higher baseline values allow more room for improvement. This underscores the potential clinical utility of using ECV and iECV quantification as non-invasive imaging biomarkers to monitor myocardial fibrosis and the response to treatment for HOCM. In fact, ECV and iECV have been used to track fibrosis changes and assess responses to interventions in mitral regurgitation, aortic regurgitation, and aortic stenosis [14], [16], [18], [19]. They are also important from a prognostic perspective. Recent reports have shown that ECV is a strong imaging marker for predicting adverse outcomes in HCM [21], [22]. Furthermore, ECV and iECV were linked to aortic stenosis progression, LV decompensation and adverse outcomes [13], [15]. Notably, iECV has been demonstrated to be more closely associated with adverse outcomes in aortic regurgitation and heart failure than LGE and even ECV, as it more accurately reflects the total LV interstitial fibrosis burden [12], [17].

Although LV mass and iECV show significant regression early following myectomy, myocardial normalization is not always possible after myectomy. Certain markers associated with irreversible changes, such as excessive LV hypertrophy [32], [33], LGE [34], [35], and increased levels of NT-proBNP [36], [37], have been shown to predict adverse outcomes. Our data reveal that focal fibrosis, as assessed by LGE, did not decrease 6 months after myectomy in HOCM patients, which aligns with those reported by Tang et al., indicating that septal myectomy may not prompt regression of LGE [4]. This is not surprising, as Rudolph et al. have previously found an absence of correlation between LGE and obstruction [38]. Intriguingly, prior studies have demonstrated that the focal myocardial fibrosis detected by LGE is closely link with pathogenic mutations in HCM, supporting the hypothesis that LGE may be genetically determined [38], [39]. Although septal myectomy can correct the hemodynamic changes in HOCM patients, it cannot alter the genetic mutations [4]. Nevertheless, it should be noted that a subset of HCM patients have negative genetic test results, suggesting unknown mutations or environmental factors may contribute, particularly in non-familial cases. Further research is necessary to explore their impact on HCM pathogenesis. Monitoring myocardial health and determining the optimal timing for intervention is thus crucial. Modern CMR imaging technology can aid in tracking myocardial structural changes, including focal and diffuse fibrosis, as well as their functional consequences. Further observations are needed to determine the future course of these LV changes and their impact on the prognosis of HOCM patients, which can aid in risk stratification and potentially optimize therapeutic management.

Notably, therapeutic options for HOCM are continuously expanding. Myosin inhibitors, including Mavacamten and Aficamten, are emerging as promising pharmacotherapies [40], [41]. Randomized trials have demonstrated their effectiveness in reducing LVOT gradients and improving symptoms [40], [41], [42]. Additionally, Mavacamten has been shown to reverse or prevent left ventricular hypertrophy, cardiomyocyte disarray, and myocardial fibrosis in animal models [43], [44]. While long-term safety and efficacy are still under evaluation, myosin inhibitors offer a hopeful future for HOCM treatment.

## 5. Limitations

Our study has limitations. First, this was a single-center study with a relatively small sample size and short follow-up duration. Given that the sample size may limit the ability to detect subtle effects or interactions, the results of this study, although valuable, should be considered hypothesis-generating and exploratory. Larger cohorts should be studied to confirm our findings, and further longitudinal studies are needed to validate the impact of iECV on long-term outcomes in HCM patients. Stepwise regression may introduce biases, such as overfitting or excluding relevant variables due to collinearity. Alternative methods, such as penalized regression, could be considered in future studies to address these issues. Additionally, the population in the present study might not entirely represent all HOCM patients due to relatively strict selection criteria. Nevertheless, our study is the first prospective analysis of postoperative diffuse myocardial fibrosis in HOCM. Moreover, we noted an increase in the extent of LGE 6 months after septal myectomy. However, since we only compared one postoperative result with the preoperative results, it is uncertain whether the increase in LGE resulted from the myectomy-related subendocardial scar or natural pathological progression. Therefore, it would be of great value to conduct a comparative analysis with a group of HCM patients who have not undergone surgical intervention, and further follow-up is essential to determine the underlying causes of the observed changes. Finally, although further research is required, it is essential to acknowledge the complexity of myocardial remodeling, which may be influenced by various factors.

## 6. Conclusions

Six months after septal myectomy, both cellular hypertrophy and diffuse fibrosis are reversible in patients with HOCM, while focal fibrosis does not regress. These changes are accompanied by the improvement of exercise parameters and laboratory biomarkers, unfolding the plastic nature of diffuse fibrosis in HOCM and its potential as a therapeutic target. Of note, the long-term dynamics of cellular hypertrophy, diffuse fibrosis, and focal fibrosis changes should be explored to assess the durability of reversibility or irreversibility.

## Funding

This work was supported by the 10.13039/501100001809National Natural Science Foundation of China (Grant No. 82371903 and 82102001), Guangdong Provincial Key Laboratory of Artificial Intelligence in Medical Image Analysis Application (No. 2022B1212010011), Guangdong Basic and Applied Basic Research Foundation (No. 2024A1515012087), and Guangzhou Clinical High-tech, Major, and Distinctive Technology Projects (2023P-TS43).

## Author contributions

Guanyu Lu drafted the manuscript. Guanyu Lu and Liqi Cao provided a method for analyzing cardiac MRI data. Jiehao Ou and Xinyi Luo acquired and analyzed the cardiac MRI data. Wei Zhu acquired and analyzed the echocardiography data. Jian Liu, Yuelong Yang, Xinyue Zhang, Peijian Wei, and Hongxiang Wu acquired and analyzed the clinical data. Zhicheng Du and Hui Liu participated in the statistical analysis and interpretation of data. Hui Liu and Huiming Guo revised the manuscript.

## Ethics approval and consent

This study was approved by the Institute Review Board of Guangdong Provincial People’s Hospital, Guangdong Academy of Medical Sciences, Guangzhou, China.

## Consent for publication

Not applicable.

## Declaration of competing interests

The authors declare that they have no competing interests.

## Data Availability

The datasets used and/or analyzed in the present study are available from the corresponding author upon reasonable request.

## Footnotes

Appendix A Supplementary data associated with this article can be found in the online version at doi:10.1016/j.jocmr.2025.101864.
