# Supplementary material for: Left ventricle myocardial remodeling following septal myectomy in patients with hypertrophic obstructive cardiomyopathy
Source: J Cardiovasc Magn Reson. 2025 Feb 17;27(1):101864. doi: 10.1016/j.jocmr.2025.101864 (PMC12022643; doi:10.1016/j.jocmr.2025.101864)
Supplement: Supplementary file 1 — Supplementary material [file mmc1.docx]

**Additional file**

**Table S1: Univariate Predictors of Change in Indexed Left Ventricular Mass after Septal Myectomy**

| Domain | Parameter | Beta | 95%CI | *P*-value |
| --- | --- | --- | --- | --- |
| Clinical | Age, yrs | 0.03 | -0.15 to 0.22 | 0.733 |
|  | Female | -0.76 | -5.98 to 4.45 | 0.770 |
|  | Body mass index, kg/m^2^ | 0.037 | -0.79 to 0.86 | 0.927 |
|  | Actual amount of resection, g | 0.14 | -0.82 to 1.10 | 0.775 |
|  | History of tobacco use | 3.69 | -6.47 to 13.85 | 0.734 |
|  | Family history of HCM | -5.24 | -14.05 to 3.58 | 0.237 |
|  | Hypertension | -0.12 | -5.93 to 5.69 | 0.967 |
|  | Diabetes | 4.24 | -5.90 to 14.37 | 0.403 |
|  | Hyperlipidemia | 3.01 | -5.06 to 11.07 | 0.456 |
|  | Atrial fibrillation | -9.15 | -21.17 to 2.87 | 0.132 |
|  | Dyspnea | 3.05 | -2.33 to 8.43 | 0.259 |
|  | Syncope | -8.48 | -18.25 to 1.39 | 0.090 |
|  | Palpitation | 0.48 | -5.19 to 6.15 | 0.864 |
|  | Chest tightness | -0.74 | -5.98 to 4.50 | 0.777 |
|  | Chest pain | 1.79 | -3.51 to 7.08 | 0.499 |
|  | 6-min walk test, m | -0.01 | -0.05 to 0.04 | 0.814 |
|  | ACE inhibitor/ARB | 10.24 | -1.70 to 22.18 | 0.091 |
|  | Beta-blocker | -4.79 | -9.96 to 0.38 | 0.069 |
|  | Calcium channel blockers | -0.18 | -6.34 to 5.99 | 0.954 |
|  | Amiodarone | -1.20 | -11.41 to 9.02 | 0.814 |
|  | Statin | 5.94 | -6.29 to 18.16 | 0.332 |
|  | Moderate or severe mitral regurgitation | 0.64 | -2.40 to 3.66 | 0.674 |
|  | SAM | 6.42 | -0.34 to 13.17 | 0.062 |
|  | Peak LVOT PG, mmHg | 0.10 | -0.04 to 0.24 | 0.146 |
|  | LVOT Vmax, m/s | 2.07 | -1.34 to 5.48 | 0.227 |
| CMR | Maximal LV wall thickness, mm | -0.43 | -0.98 to 0.11 | 0.113 |
|  | LVEDVI, ml/m^2^ | 0.12 | -0.03 to 0.27 | 0.108 |
|  | LVESVI, ml/m^2^ | 0.15 | -0.11 to 0.41 | 0.259 |
|  | SVI, ml/m^2^ | 0.16 | -0.07 to 0.38 | 0.161 |
|  | LVEF, % | -0.05 | -0.50 to 0.40 | 0.835 |
|  | LVMI, g/m^2^ | 0.08 | 0.003 to 0.161 | **0.041** |
|  | Cardiac output, L/min | 1.67 | -0.21 to 3.54 | 0.080 |
|  | LGE 6SD method, g | -0.07 | -0.25 to 0.11 | 0.419 |
|  | LGE 6SD method, g/m^2^ | -0.11 | -0.41 to 0.19 | 0.460 |
|  | LGE 6SD method, % | -0.43 | -0.89 to 0.03 | 0.067 |
|  | Native T1, ms | -0.07 | -0.14 to 0.01 | 0.079 |
|  | ECV, % | -1.13 | -1.83 to -0.43 | **0.002** |
|  | iCV, mL/m^2^ | 0.20 | 0.08 to 0.33 | **0.002** |
|  | iECV, mL/m^2^ | 0.25 | -0.11 to 0.61 | 0.161 |
| Blood | NT-proBNP, pg/mL | 0.70 | -2.16 to 3.55 | 0.625 |
|  | hs-cTNT, pg/mL | -0.49 | -4.19 to 3.21 | 0.791 |

Bold p values indicate statistical significance.

Change in LVMI = Preoperative LVMI - Postoperative LVMI. All predictors reflect baseline (preoperative) data or measurements.

ACE = angiotensin-converting-enzyme; ARB = angiotensin receptor blocker; CMR = cardiac magnetic resonance; CI = confidence interval; DBP = diastolic blood pressure; ECV = extracellular volume fraction; hs-cTnT = high-sensitivity cardiac troponin T; HCM = hypertrophic cardiomyopathy; iCV = indexed cellular volume; iECV = indexed extracellular volume; LGE = late gadolinium enhancement; LV = left ventricular; LVEDVI = left ventricular end-diastolic volume index; LVEF = left ventricular ejection fraction; LVESVI = left ventricular end-systolic volume index; LVMI = left ventricular mass index; LVOT = left ventricular outflow tract; NT-proBNP = N-terminal pro–B-type natriuretic peptide; PG = pressure gradient; SAM = systolic anterior motion; SBP = systolic blood pressure; SD = standard deviation; SVI = stroke volume index; Vmax = peak velocity.

**Table S2: Univariate Predictors of Change in Indexed Extracellular Volume after Septal Myectomy**

| Domain | Parameter | Beta | 95%CI | *P*-value |
| --- | --- | --- | --- | --- |
| Clinical | Age, yrs | 0.01 | -0.05 to 0.07 | 0.776 |
|  | Female | -0.53 | -2.15 to 1.09 | 0.511 |
|  | Body mass index, kg/m^2^ | 0.097 | -0.16 to 0.35 | 0.448 |
|  | Actual amount of resection, g | 0.09 | -0.21 to 0.39 | 0.551 |
|  | History of tobacco use | 2.87 | -0.19 to 5.92 | 0.065 |
|  | Family history of HCM | 1.64 | -1.11 to 4.38 | 0.235 |
|  | Hypertension | 1.43 | -0.327 to 3.177 | 0.108 |
|  | Diabetes | 2.07 | -1.05 to 5.19 | 0.188 |
|  | Hyperlipidemia | 0.02 | -2.33 to 2.73 | 0.873 |
|  | Atrial fibrillation | 0.65 | -3.20 to 4.50 | 0.341 |
|  | Dyspnea | 0.51 | -1.19 to 2.20 | 0.550 |
|  | Syncope | -1.31 | -4.47 to 1.85 | 0.406 |
|  | Palpitation | -0.49 | -2.25 to 1.27 | 0.580 |
|  | Chest tightness | 0.05 | -1.59 to 1.68 | 0.953 |
|  | Chest pain | 0.46 | -1.19 to 2.11 | 0.577 |
|  | 6-min walk test, m | 0.00 | -0.01 to 0.01 | 0.976 |
|  | ACE inhibitor/ARB | 2.79 | -0.96 to 6.54 | 0.141 |
|  | Beta-blocker | -0.46 | -2.13 to 1.22 | 0.584 |
|  | Calcium channel blockers | 1.22 | -0.66 to 3.11 | 0.197 |
|  | Amiodarone | -2.90 | -5.95 to 0.15 | 0.062 |
|  | Statin | 0.41 | -3.44 to 4.26 | 0.831 |
|  | Moderate or severe mitral regurgitation | -0.88 | -1.78 to 0.03 | 0.058 |
|  | SAM | -1.14 | -3.31 to 1.03 | 0.294 |
|  | Peak LVOT PG, mmHg | -0.12 | -0.06 to 0.03 | 0.400 |
|  | LVOT Vmax, m/s | -0.69 | -1.75 to 0.37 | 0.198 |
| CMR | Maximal LV wall thickness, mm | 0.02 | -0.16 to 0.19 | 0.853 |
|  | LVEDVI, ml/m^2^ | 0.01 | -0.04 to 0.06 | 0.702 |
|  | LVESVI, ml/m^2^ | 0.05 | -0.04 to 0.13 | 0.267 |
|  | SVI, ml/m^2^ | -0.02 | -0.09 to 0.05 | 0.556 |
|  | LVEF, % | -0.11 | -0.25 to 0.03 | 0.107 |
|  | LVMI, g/m^2^ | 0.02 | -0.003 to 0.047 | 0.087 |
|  | Cardiac output, L/min | 0.36 | -0.24 to 0.96 | 0.228 |
|  | LGE 6SD method, g | 0.02 | -0.04 to 0.07 | 0.514 |
|  | LGE 6SD method, g/m^2^ | 0.04 | -0.06 to 0.13 | 0.434 |
|  | LGE 6SD method, % | 0.02 | -0.13 to 0.17 | 0.805 |
|  | MVO | -0.99 | -3.05 to 1.08 | 0.341 |
|  | Native T1, ms | 0.02 | -0.01 to 0.04 | 0.213 |
|  | ECV, % | 0.27 | 0.04 to 0.50 | **0.024** |
|  | iCV, mL/m^2^ | 0.03 | -0.01 to 0.07 | 0.180 |
|  | iECV, mL/m^2^ | 0.15 | 0.05 to 0.26 | **0.006** |
| Blood | NT-proBNP, pg/mL | 0.16 | -0.74 to 1.04 | 0.727 |
|  | hs-cTNT, pg/mL | 0.57 | -0.57 to 1.71 | 0.319 |

Bold p values indicate statistical significance.

Change in iECV = Preoperative iECV - Postoperative iECV. All predictors reflect baseline (preoperative) data or measurements.

The abbreviations as in Table S1.

**Table S3: Predictors of Change in Indexed Left Ventricular Mass and Indexed Extracellular Volume After Septal Myectomy (without adjustment for baseline markers)**

|  | Univariable Predictors | | | Multivariable Predictors | | |
| --- | --- | --- | --- | --- | --- | --- |
|  | Beta | 95%CI | *P*-value | Beta | 95%CI | *P*-value |
| Predictors of change in LVMI after Septal Myectomy | | | | | | |
| Age, yrs | 0.03 | -0.15 to 0.22 | 0.733 |  |  |  |
| Female | -0.76 | -5.98 to 4.45 | 0.770 |  |  |  |
| Actual amount of resection, g | 0.14 | -0.82 to 1.10 | 0.775 |  |  |  |
| LGE 6SD method, % | -0.43 | -0.89 to 0.03 | 0.067 |  |  |  |
| Native T1, ms | -0.07 | -0.14 to 0.01 | 0.079 |  |  |  |
| ECV, % | -1.13 | -1.83 to -0.43 | 0.002 | -0.78 | -1.52 to -0.04 | 0.040 |
| iCV, mL/m^2^ | 0.20 | 0.08 to 0.33 | 0.002 | 0.15 | 0.02 to 0.27 | 0.030 |
| iECV, mL/m^2^ | 0.25 | -0.11 to 0.61 | 0.161 |  |  |  |
| Predictors of change in iECV after Septal Myectomy | | | | | | |
| Age, yrs | 0.01 | -0.05 to 0.07 | 0.776 |  |  |  |
| Female | -0.53 | -2.15 to 1.09 | 0.511 |  |  |  |
| Actual amount of resection, g | 0.09 | -0.21 to 0.39 | 0.551 |  |  |  |
| LVMI, g/m^2^ | 0.02 | -0.003 to 0.047 | 0.087 |  |  |  |
| LGE 6SD method, % | 0.02 | -0.13 to 0.17 | 0.805 |  |  |  |
| Native T1, ms | 0.02 | -0.01 to 0.04 | 0.213 |  |  |  |
| ECV, % | 0.27 | 0.04 to 0.50 | 0.024 |  |  |  |
| iCV, mL/m^2^ | 0.03 | -0.01 to 0.07 | 0.180 |  |  |  |

Change in LVMI or iECV = preoperative LVMI or iECV - postoperative LVMI or iECV. All predictors reflect baseline (preoperative) data or measurements.

The abbreviations as in Supplemental Table S1.


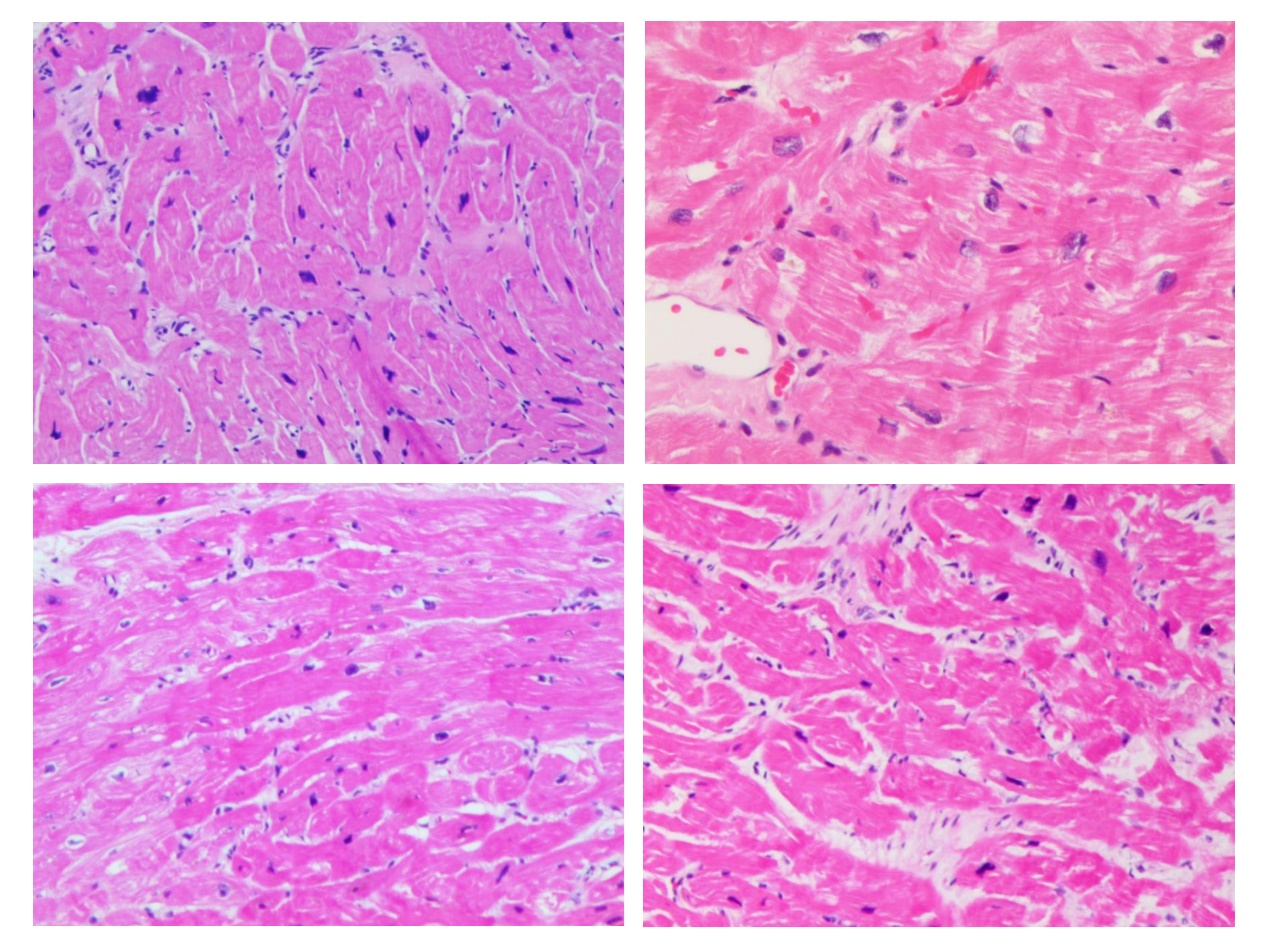
Figure S1: The histological sample from 4 HOCM specimens revealed myocyte hypertrophy and disarray (hematoxylin and eosin staining). HOCM = hypertrophic obstructive cardiomyopathy.


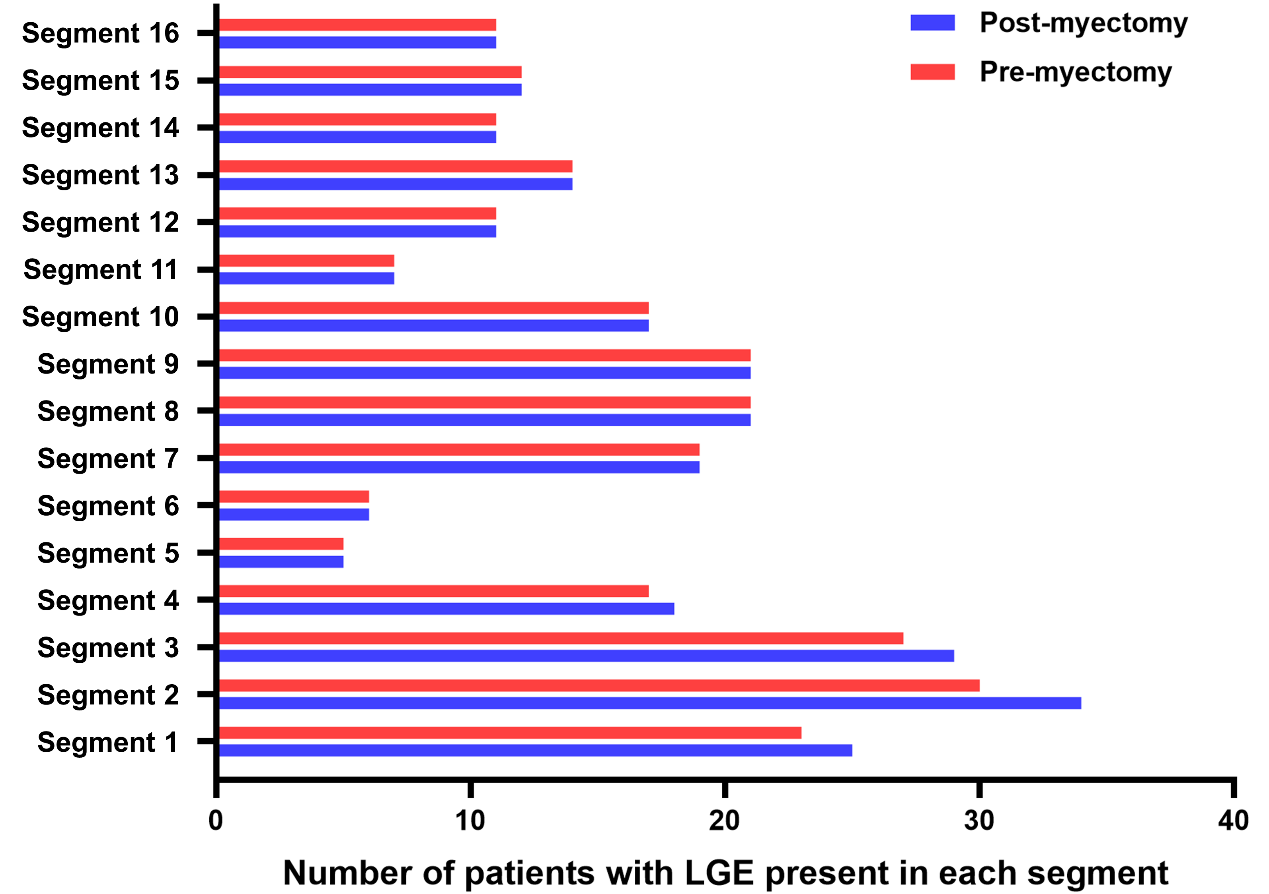
Figure S2: LGE location in 43 participants with hypertrophic obstructive cardiomyopathy pre- and post-myectomy. The histogram shows the number of patients with LGE in each segment. LGE = late gadolinium enhancement.


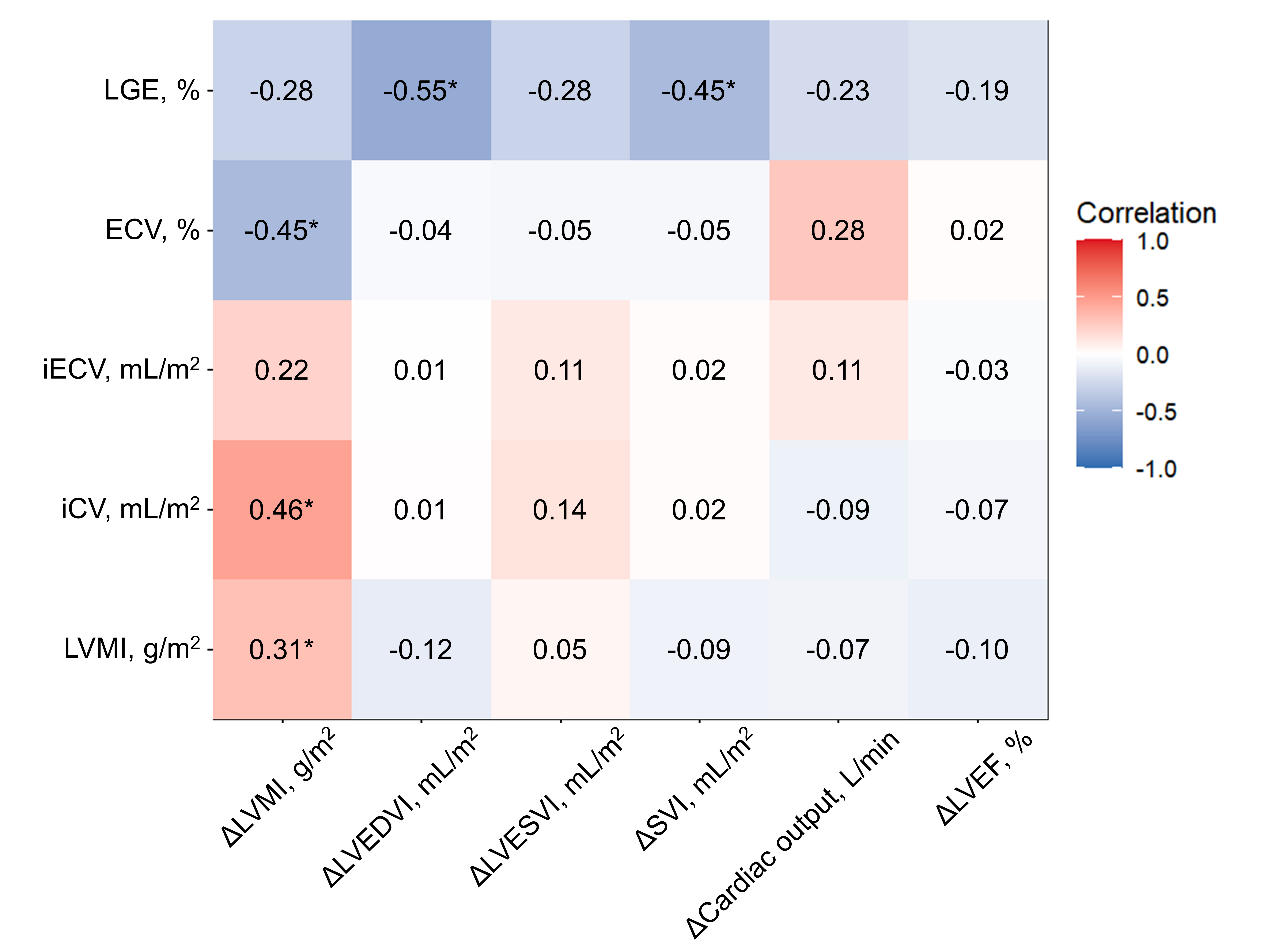


Figure S3: Correlations between the fibrosis variables and changes in cardiac size and function.

Δ = preoperative value - postoperative value; The number in each grid represents the correlation coefficient (r); *indicate that the correlations are statistically significant (*P* < 0.05).

ECV = extracellular volume fraction; iCV = indexed cellular volume; iECV = indexed extracellular volume; LGE = late gadolinium enhancement; LVEDVI = left ventricular end-diastolic volume index; LVEF = left ventricular ejection fraction; LVESVI = left ventricular end-systolic volume index; LVMI = left ventricular mass index; SVI = stroke volume index.


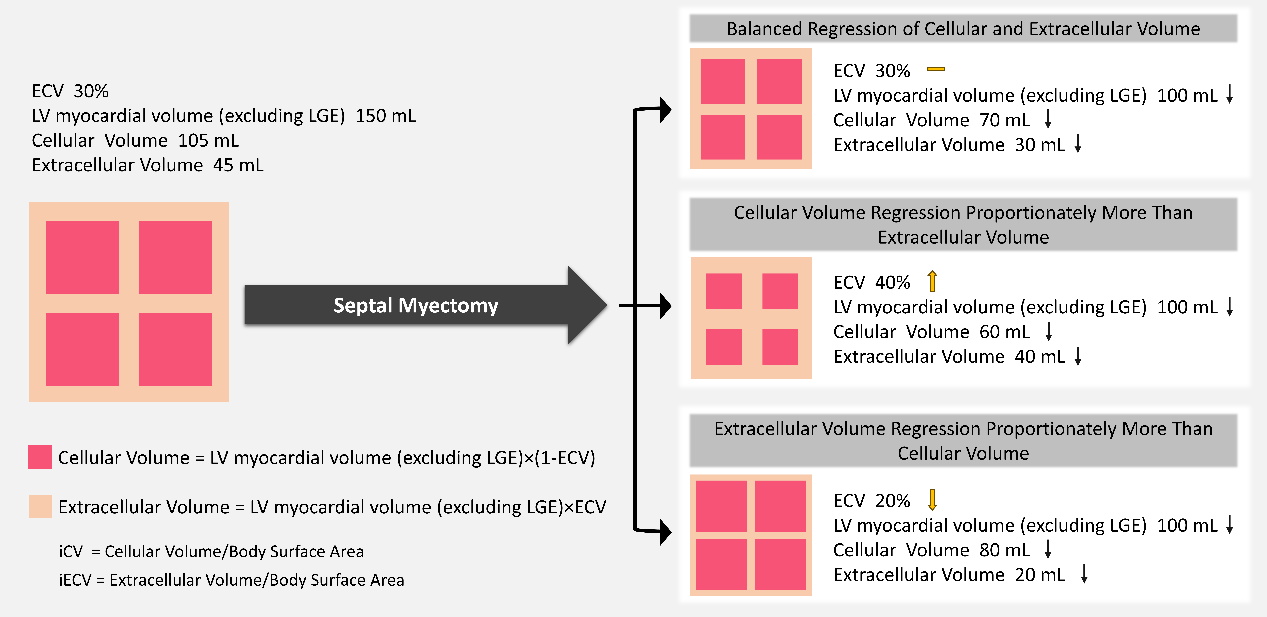


Figure S4: ECV dichotomizes the myocardium into cellular and extracellular compartments.

ECV = extracellular volume fraction; iCV = indexed cellular volume; iECV = indexed extracellular volume; LGE = late gadolinium enhancement; LV = left ventricular.
